# Efficient Algorithms towards Network Intervention

**DOI:** 10.1145/3366423.3380269

**Published:** 2020-04

**Authors:** Hui-Ju Hung, Chih-Ya Shen, Wang-Chien Lee, Zhen Lei, De-Nian Yang, Sy-Miin Chow

**Affiliations:** The Pennsylvania State Univ., USA; National Tsing Hua Univ., Taiwan; The Pennsylvania State Univ., USA; The Pennsylvania State Univ., USA; Academia Sinica, Taiwan; The Pennsylvania State Univ., USA

**Keywords:** Network intervention, optimization algorithms, social networks

## Abstract

Research suggests that social relationships have substantial impacts on individuals’ health outcomes. Network intervention, through careful planning, can assist a network of users to build healthy relationships. However, most previous work is not designed to assist such planning by carefully examining and improving multiple network characteristics. In this paper, we propose and evaluate algorithms that facilitate network intervention planning through simultaneous optimization of network *degree, closeness, betweenness,* and *local clustering coefficient,* under scenarios involving *Network Intervention with Limited Degradation - for Single target (NILD-S)* and *Network Intervention with Limited Degradation - for Multiple targets (NILD-M).* We prove that NILD-S and NILD-M are NP-hard and cannot be approximated within any ratio in polynomial time unless P=NP. We propose the *Candidate Re-selection with Preserved Dependency (CRPD)* algorithm for NILD-S, and the *Objective-aware Intervention edge Selection and Adjustment (OISA)* algorithm for NILD-M. Various pruning strategies are designed to boost the efficiency of the proposed algorithms. Extensive experiments on various real social networks collected from public schools and Web and an empirical study are conducted to show that CRPD and OISA outperform the baselines in both efficiency and effectiveness.

## 1 INTRODUCTION

Previous studies have shown the importance and strengths of social relationships in influencing individual behaviors. Strong social relationships have been shown to facilitate the dissemination of information, encourage innovations, and promote positive behavior [43]. Also, social relationships surrounding individuals have substantial impacts on individuals’ mental and physical health [12, 17]. For example, studies in Science and American Sociological Review indicate that socially isolated individuals are more inclined to have mental health problems and physical diseases, ranging from psychiatric disorders to tuberculosis, suicide, and accidents [13, 17].

To alleviate the problems that arise with social isolation, two classes of intervention strategies may be adopted, including: 1) *personal intervention*, which guides individuals to understand their situations, attitudes, and capacities through counseling [9]; and 2) *network intervention*, which emphasizes the need to strengthen individuals’ social networks to accelerate behavior changes that can lead to desirable outcomes at the individual, community, or organizational level [12, 17]. As an example, network intervention that helps establish new social links is crucial for individuals with autism spectrum disorders [6]. Those new links may be effectively introduced by curative groupings and network meetings – events which encourage them to socialize more frequently [11, 14].

In order for network intervention to improve individual health outcomes, it is crucial to add social links in ways that promote network characteristics found to be related to positive outcomes. Thus, an important question to ask is: given an individual, what properties define the strength of the individual’s network? Previous studies point out several possibilities: 1) *Degree* indicates the number of established friendships. An individual with a large degree is more popular and has more opportunities to establish self-identity and social skills [42]. Thus, a large-degree individual is less inclined to be socially-isolated and have mental health problems [13]. 2) *Closeness* indicates the inverse of the average social distance from an individual to all others in the network. An individual with great closeness is typically located in the center of the network and tend to perceive a lower level of stress [18]. 3) *Betweenness* indicates the tendency of an individual to fall on the shortest path between pairs of other individuals. An individual with large betweenness tends to occupy *brokerage* positions in the network and have more knowledge of events happening in the network. Thus, an individual with large betweenness may also perceive social relationships more accurately [23]. 4) Finally, *Local clustering coefficient* *(LCC)* indicates the diversity of relationships within an individual’s ego network [12]. Specifically, an individual with a small LCC tends to have diverse relationships since her friends are less likely to be acquainted with each other [12]. The perspective that individuals with small LCCs are less likely to have mental health problems has been postulated in the functional specificity theory, which advocates the need for having different support groups for distinct functions e.g., by obtaining *attachment* from families or friends, *social integration* from social activity groups, and *guidance* from colleagues [49]. In other words, individuals with large LCCs tend not to build a diverse social network by putting all their relationship eggs in a few baskets, and tend to have depressive symptoms and neurological illnesses [12]. This relationship has been validated in studies with participants across various cultures and ages. In a study involving 173 retired US elders, higher LCC is found to be associated with lower life satisfaction, self-esteem, happiness, and higher depression [51]. In another study involving 2844 high school students, higher LCC again is associated with lower self-esteem [41].

In practice, specialists and practitioners may not have the time or resources to provide frequent and ongoing relationship recommendations to every individual. Also, the recommendations made by persons are susceptible to their subjective biases. As such, supplemental, objective information from automated network planning algorithms that can simultaneously optimize multiple network characteristics are helpful and valuable. This paper aims to develop novel algorithms that recommend suitable intervention links based on multiple potentially health-enhancing network characteristics. However, adding social links is not always straightforward for network characteristics. Of the characteristics noted earlier, the degree for each individual can be improved by adding more edges, in which case the closeness and betweenness of the network are also enhanced. However, improving the LCC is more challenging as the LCCs of individuals and nearby friends may not always improve, and they can even deteriorate, when more edges are added.

**Figure 1:**
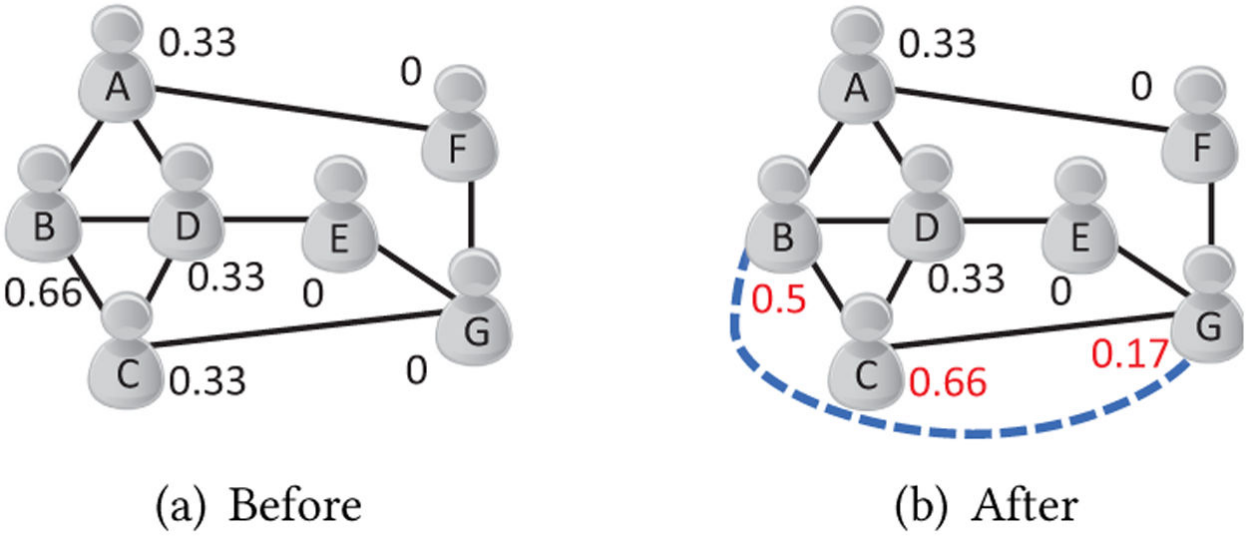
An illustration on challenges of intervention

Selecting good intervention links based on the LCC is further deterred by other challenges. A new link established for a targeted individual may increase the LCCs of her friends, when those new friends are acquainted with each other. Figure 1 presents an example showing the side-effects of adding improper new links on the LCC. Figure 1(a) shows a social network in which the nodes are annotated by their initial LCCs, and Figure 1(b) presents the network after adding an edge from B to G. The LCC of B is effectively reduced to 0.5, but the LCC of C unfortunately grows to 0.66. The example shows that heuristic or uninformed selection of intervention links by specialists and practitioners may lead to undesirable changes in the LCC; even worse, the undesirable changes may happen to many nearby individuals when the network size is large. Moreover, even though a simple way to decrease LCC is to remove some existing social links, this approach is *not* considered because removal of existing social links undermines established social support [40].

In this paper, we propose and test several algorithms that can simultaneously optimize network LCC, closeness, betweenness, and degree. We first formulate a new problem, namely, *Network Intervention with Limited Degradation - for Single target (NILD-S)*. Given a budget of *k*, a threshold *τ*, and a target *t*, NILD-S finds the set *F* of *k* intervention edges to minimize the LCC of *t*, such that the side effect, in terms of increment in anyone’s LCC, cannot exceed *τ*. NILD-S also ensures that the degree, betweenness and closeness of *t* exceed given thresholds. We propose *Candidate Re-selection with Preserved Dependency* (CRPD) algorithm, which first obtains an initial solution by extracting the individuals with the smallest degrees, and improves the initial solution by re-examining the candidates filtered out by nodes involved in the solution. Note that CRPD selects edges according to multiple characteristics. We prove that NILD-S is NP-hard and cannot be approximated within any ratio in polynomial time unless P=NP. Nevertheless, we prove that CRPD can find the optimal solution for *threshold graphs*, which are very similar to many well-known online social networks regarding many measurements like degree distribution, diameter and clustering coefficient [26, 31, 45].

Finally, we seek to extend the NILD-S to simultaneously improve the LCCs of multiple individuals while ensuring other network characteristics, including betweenness, closeness, and degree. To do so, we formulate the *Network Intervention with Limited Degradation - for Multiple targets (NILD-M)* problem to jointly minimize the LCCs of multiple targets. Given the aforementioned *k*, *τ*, and the set of targets *T*, NILD-M finds the set *F* of *k* intervention edges such that the maximal LCC of individuals in *T* is minimized, while the LCC increment of any person does not exceed *τ*. NILD-M also ensures that the degree, betweenness, and closeness of all targetes exceed their minimum thresholds. We prove that NILD-M is NP-hard and cannot be approximated within any ratio in polynomial time unless P=NP. To solve NILD-M, we design *Objective-aware Intervention edge Selection and Adjustment (OISA)*, which 1) carefully examines both the LCC of each terminal and the network structure to ensure the constraint of *τ*, 2) explores the idea of *optionality* to improve the solution quality, and 3) derives the lower bound on the number of required edges and the LCC upper bounds to effectively reduce computational time. Also, we evaluate OISA via an empirical study on four psychological outcomes, *anxiety*, *perceived stress, positive and negative emotions*, and *psychological well-being*.

The contributions are summarized as follows.

- Previous research has suggested the use of network intervention in improving health outcomes. With the potential to increase the support network by new acquaintances, however, there is no effective planning tool for practitioners to select suitable intervention edges. We formulate NILD-S and NILD-M to address this critical need for identifying suitable intervention links for a single target and a group of targets, while considering multiple network characteristics.
- We prove that NILD-S is NP-hard and cannot be approximated within any ratio in polynomial time unless P=NP. We propose CRPD and prove that CRPD obtains the optimal solution for threshold graphs.
- We prove that NILD-M is NP-hard and cannot be approximated within any ratio in polynomial time unless P=NP and design OISA for NILD-M.
- Experiments on real datasets show that the proposed CRPD and OISA efficiently find near-optimal solutions for NILD-S and NILD-M and outperform the baselines. Also, an empirical study assessed by clinical psychologists and professors in the field manifests that the network intervention alleviates self-reported health outcomes of participants, and the effects are statistically significant over another control group.

The rest of this paper is organized as follows. Section 2 reviews related work. Section 3 formulates NILD-S, analyzes its theoretical hardness, and proposes CRPD. Section 4 formulates NILD-M and analyzes its theoretical hardness. Section 5 proposes OISA. Section 6 reports the experiments. Finally, Section 7 concludes the paper.

## 2 RELATED WORK

The theory of network intervention has been studied in the fields of psychology, behavioral health, and education for lowering negative emotions by enhancing social integration, support, engagement, and attachment [3]. It has also been adopted for family therapy and bullying avoidance [14]. In education, network intervention has been implemented to facilitate knowledge dissemination among students, thereby improving student learning [44]. Under current practice, new intervention links are typically selected heuristically by practitioners [22, 34]. However, it is very challenging to consider multiple persons simultaneously without deteriorating the status of surrounding individuals. Thus, it would be worthwhile to develop algorithms for this important need.

In the field of social network analysis, researchers have paid considerable attention to efficiently finding the number of triangles [24, 27] and selecting a group of individuals with the maximum or minimum number of triangles [33]. Notice that the above-mentioned research mostly focuses on measuring structural properties of nodes in static or dynamic networks, with no intention to tailor and change the network graph. Recently, a new line of research in network science has emerged with the objective of revising a network graph according to specific network characteristics. These include maximizing the closeness centrality, betweenness centrality and influence score, minimizing the diameter, and enhancing the network robustness [10, 29, 50]. However, these algorithms do not include LCC as a target network characteristic for intervention purposes. Importantly, none of these algorithms was designed to optimize multiple network characteristics simultaneously.

Recently, owing to the success of online social networks, reported cases of social network mental disorders have increased, motivating new collaborations between data scientists and mental health practitioners. New machine learning frameworks have been shown to be helpful in identifying patients tending to be vulnerable, and even have clinical levels of negative emotions and unhealthy living [30, 35-37]. However, those are not designed for network intervention, which actually changes the network graph. Finally, link prediction [1, 4, 7, 15, 32, 47, 52] has been widely studied. Existing algorithms usually recommend individuals sharing many common friends and similar interests to become friends. However, they are not designed for network intervention, which does not necessarily prefer people socially close or with similar backgrounds.

## 3 INTERVENTION FOR A SINGLE TARGET

In this section, we first reduce the *Local Clustering Coefficient (LCC)* of a targeted individual (denoted as *t*) by selecting a set of people from the social network to become friends with *t*. Given a social network *G* = (*V*, *E*) (or *G* for short), where each node *v* ∈ *V* denotes an individual, and each edge (*i*, *j*) ∈ *E* represents the social link between individuals *i* and *j*, the *ego network* of an individual *v* is the subgraph induced by *v* and its neighbors *N_G_*(*v*). The LCC of a node *v* in *G*, *LCC_G_*(*v*), is defined as the number of edges between the nodes in *N_G_*(*v*) divided by the maximum number of possible edges among the nodes in *N_G_*(*v*),

(1) $$LCC_{G}(v)=\frac{|\{(i,j)|i,j\in N_{G}(v),(i,j)\in E\}|}{C(d_{G}(v),2)}$$

where *d_G_*(*v*) = ∣*N_G_*(*v*)∣ and *C*(*d_G_*(*v*), 2) is the number of combinations to choose two items from *d_G_*(*v*) ones. Adding social links may increase LCC of other nodes not incident to any new edge.

However, the increment of LCC for healthy people also needs to be carefully controlled.^1^ In addition to LCC, it is also important to ensure that the degree, betweenness, and closeness are sufficiently large. Therefore, we formulate the *Network Intervention with Limited Degradation - for Single target* (NILD-S) problem as follows.

Definition 1. *Given a social network G* = (*V*, *E*) *(or G for short), the target t, the number k of intervention edges to be added, the LCC degradation threshold τ, the lower bounds on betweenness, closeness and degree* *ω_b_*, *ω_c_*, *and ω*_*d*_, *NILD-S minimizes the LCC of t by adding a set F of k edges incident to t, such that in the new network* $\bar{G}$, *1)* $LCC_{\bar{G}}(v)-LCC_{G}(v)\leq \tau$ *for any v, 2)* $b_{\bar{G}}(t)>\omega_{b}$, *3)* $c_{\bar{G}}(t)>\omega_{c}$, *and 4)* $d_{\bar{G}}(t)>\omega_{d}$, *where* $b_{\bar{G}}(t)$, $c_{\bar{G}}(t)$ *and* $d_{\bar{G}}(t)$ *are the betweenness, closeness, and degree of t in* $\bar{G}$.

NILD-S is computationally expensive. We prove that it is NP-hard and inapproximable within any ratio, i.e., there is no approximation algorithm with a finite ratio for NILD-S unless P=NP. However, later we show that NILD-S is tractable for threshold graphs, which share similar graph properties with many well-known online social networks, e.g., Live-Journal, Flickr, and Youtube [26, 31, 45].

Theorem 1. *NILD-S is NP-hard and cannot be approximated within any ratio in polynomial time unless P=NP*.

Proof. We prove the NP-hardness by the reduction from the Maximum Independent Set (MIS) problem under triangle-free graphs (i.e., a graph without any three nodes forming a triangle) [25]. Given a triangle-free graph *G_M_* = (*V_M_*, *E_M_*), MIS is to find the largest subset of nodes *S_M_* ⊆ *V_M_*, such that every node in *S_M_* is not adjacent to any other nodes in *S_M_*. For each instance of MIS, we construct an instance *G* = (*V*, *E*) of NILD-S as follows. For each node *v*′ ∈ *V_M_* and edge (*i*′, *j*′) ∈ *E_M_*, we create the corresponding node *v* ∈ *V* and edge (*i*, *j*) ∈ *E*, respectively. Also, we add a node *t* as the targeted node and set *τ*, *ω_b_*, *ω_c_*, and *ω_d_* as 0. In the following, we prove that *G_M_* = (*V_M_*, *E_M_*) has an independent set *S_M_* with size *k* in MIS if and only if the LCC of *t* in *G* = (*V*, *E*) remains as 0 after adding (*t*, *v*) for every *v*′ ∈ *S_M_*. We first prove the sufficient condition. If *G_M_* = (*V_M_*, *E_M_*) has an independent set *S_M_* of size *k*, then there is no edge between any two nodes in *S_M_*. Thus, if we add an edge (*v*, *t*) for each node *v*′ ∈ *S_M_* in *G*(*V*, *E*), the LCC of *t* is still 0. We then prove the necessary condition. If there is a set *S* of *k* nodes such that *t*’s LCC remains as 0 after adding (*v*, *t*) to *E*, then there exists no edge among *t*’s neighbors, i.e., *S*. Therefore, *S_M_* with the corresponding nodes in *S* is an independent set.

Next, we prove that NILD-S cannot be approximated within any ratio in polynomial time unless P=NP by contradiction. Assuming that there exists a polynomial-time algorithm with solution *lcc* to approximate NILD-S with a finite ratio *ro* for a triangle-free *G* = (*V*, *E*), i.e., the LCC of the optimal solution is at least *lcc/ro*. If *lcc* = 0, there is an independent set with size *k*. If *lcc* > 0, the LCC of the optimal solution is at least *lcc/ro* > 0, and there is no *k*-node independent set. Thus, the approximation algorithm for NILD-S can solve MIS in polynomial time by examining *lcc*, contradicting that MIS is NP-hard [25].

### 3.1 The CRPD Algorithm

For NILD-S, a simple approach is to iteratively choose a node *u*, add (*t*, *u*) into *F*, and eliminate (i.e., does not regard it as a candidate in the future) every neighbor *r* of *u* if adding both (*t*, *u*) and (*t*, *r*) into *F* would increase the LCC of any node for more than *τ* (called the LCC degradation constraint). However, the above approach does not carefully examine the structure among the neighbors of *t*. The selection of *u* is crucial, because it may become difficult to choose its neighbors for connection to *t* later due to the LCC degradation constraint. Therefore, a simple baseline is to extract the *u* with the smallest degree and add (*t*, *u*) to *F*, because such *u* tends to result in the least number of neighbors removed from the pool of candidates for connecting to *t*. It removes those neighbors *r* of *u* if adding (*t*, *u*) increases LCC of any individual by more than *τ*. However, Example 1 indicates that a good candidate *r* may be improperly removed due to a small LCC.

**Figure 2:**
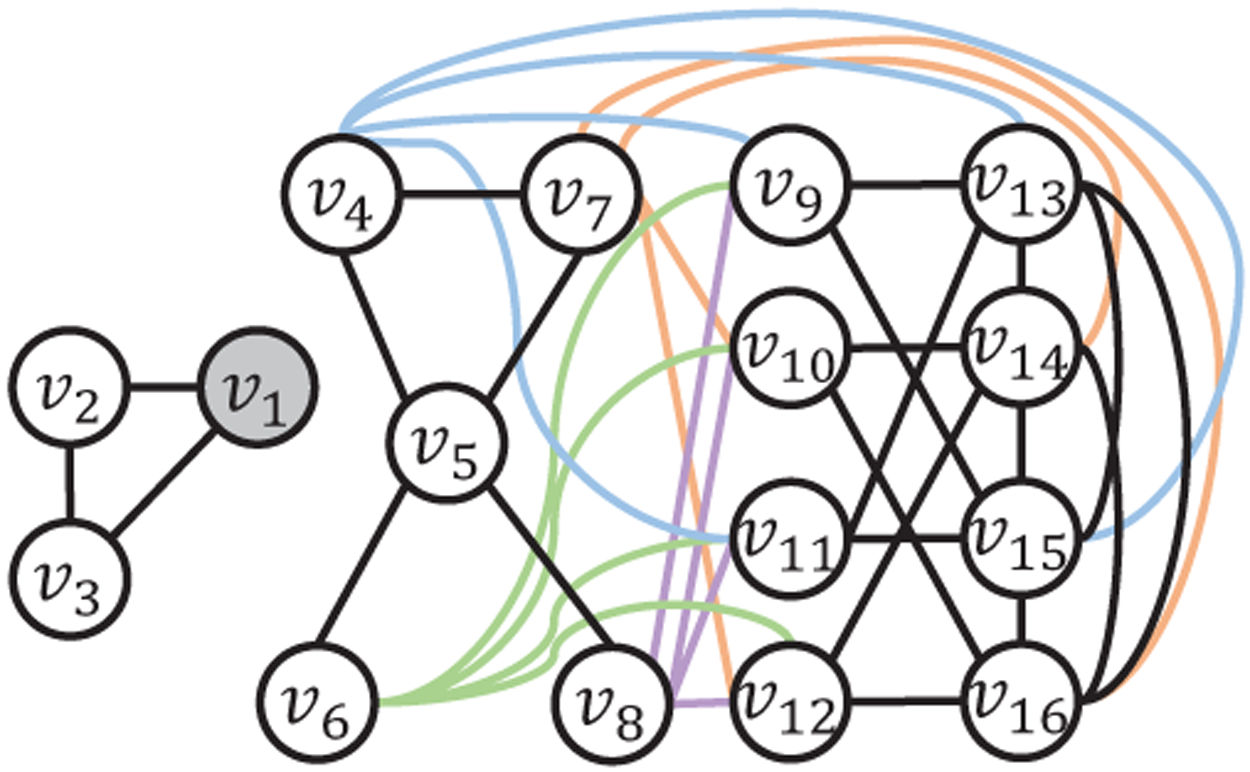
A motivating example of CRPD

Example 1. Figure 2 *shows an example of NILD-S with 16 nodes, where t* = *v*_1_, *k* = 3, *τ* = 0.05, *ω_b_* = 0.5, *ω_c_* = 0.5, and *ω_d_* = 4. *Note that all edges in* Figure 2 *are edges in E regardless of their colors. The baseline first selects v*_5_ *(i.e., adding* (*v*_1_, *v*_5_) *to F) since it has the smallest degree among all nodes not connected to v*_1_. *Then, for the neighbor V*_4_ *of v*_5_, *adding* (*v*_1_, *v*_4_) *to F* = {(*v*_1_, *v*_5_)} *does not increase LCC of any node to more than τ. Thus, V*_4_ *is still a valid candidate*.^2^ *However, after choosing v*_5_, *the baseline excludes v*_6_ *and v*_8_ *from candidates as their respective LCCs will be increased by 0.06. Afterward, the baseline selects v*_13_ *and v*_14_ *to reduce the LCC of v*_1_ *from 1 to 0.2. Nevertheless, a better approach is to select v*_6_, *v*_8_ *and V*_4_, *as the LCC of v*_1_ *can effectively diminish to 0.1*.

**Table:** 

Algorithm 1: The CRPD algorithm
$$\begin{array}{cc} & \;\mathrm{Require}:\;G,\;t,\;k,\;\tau ,\;\omega_{b},\;\omega_{c},\;\omega_{d}\\ & \;\mathrm{Ensure}:\;\text{A set}\;F\;\text{of}\;k\;\text{edges incident to}\;t\;\text{to be added}\\ & \;\;1:(F,R)\leftarrow Baseline(t,k,\emptyset )\;∕∕\text{baseline}\\ & \;\;2:\text{Retrieve the minimum degree}\;r_{m}\;\text{from}\;R\\ & \;\;3:(F^{\prime },R^{\prime })\leftarrow Baseline(t,k,\{(t,r_{m})\})\;∕∕\text{RNR}\\ & \;\;4:\text{Replace}\;F\;\text{with}\;F^{\prime }\;\text{if}\;F^{\prime }\;\text{is better}\\ & \;\;5:\mathrm{return}\;F\\ & \;\;6:\mathrm{function}\;\text{Baseline}(t,k,F)\;∕∕\text{function called by CRPD}\\ & \;\;7:\;\;\;\text{Let}\;C\;\text{include all}\;i\;\text{s.t. add}\;(t,i)\;\text{satisfies the}\;\tau \;\text{constraint}\\ & \;\;8:\;\;\;R\leftarrow \emptyset \\ & \;\;9:\;\;\;\mathrm{while}\;|F|<k\;\mathrm{do}\\ & 10:\;\;\;\;\text{Choose}\;u\;\text{from}\;C\;\text{according to}\;d_{G}(u)\;\text{and Eq.2}\\ & 11:\;\;\;\;\mathrm{if}\;\exists i\in C\;\text{s.t. add}\;(t,i)\;\text{violates the}\;\tau \;\text{constraint}\;\mathrm{them}\\ & \;12:\;\;\;\;\;C\leftarrow C\setminus \{i\};R\leftarrow R\cup \{i\}\\ & \;13:\;\mathrm{return}\;(F,R) \end{array}$$

Motivated by Example 1, we propose the *Candidate Re-selection with Preserved Dependency* (CRPD) algorithm for NILD-S. CRPD includes two components: 1) *Removed Node Re-selection Strategy*, and 2) *Multi-measurement Integration Selection Strategy*, as follows. A pseudocode of CRPD is shown in Algorithm 1.

#### Removed Node Re-selection (RNR) Strategy.

To avoid missing good candidates when processing each node *u* in the above baseline, CRPD first extracts *R* during the process, where each *r* in *R* is a neighbor of *u* removed by the baseline due to the LCC degradation constraint, i.e., including both (*t*, *u*) and (*t*, *r*) in *F* increases any node’s LCC for more than *τ*. CRPD improves the above baseline by conducting a deeper exploration that tries to replace (*t*, *u*) by (*t*, *r_m_*), where *r_m_* is the node with the minimum degree in *R*, if selecting (*t*, *r_m_*) instead removes fewer neighbors and obtains a better solution later. In Example 1, adding (*v*_1_, *v*_5_) to *F* removes its neighbors *v*_6_ and *v*_8_ because including (*v*1, *v*_6_) or (*v*_1_, *v*_8_) to *F* = {(*v*_1_, *v*_5_)} increases the LCCs of *v*_6_ or *v*_8_ by more than *τ*, respectively. In contrast, adding (*v*_1_, *v*_6_) (instead of (*v*_1_, *v*_5_)) to *F* only removes *v*_5_ and obtains a better solution {(*v*_1_, *v*_6_), (*v*_1_, *v*_8_), (*v*_1_, *v*_4_)}, compared with the solution {(*v*_1_, *v*_5_), (*v*_1_, *v*_13_), (*v*_1_, *V*_14_)} of the baseline. With the above deeper inspection, later we prove that CRPD can find the optimal solution of NILD-S in threshold graphs.

#### Multi-measurement Integration Selection Strategy (MISS).

To address *ω_d_*, the degree of *t* can be examined according to *k* + *d_G_*(*t*) when more edges are included. However, when the betweenness and closeness of *t* are smaller than *ω_b_* and *ω_c_*, respectively, CRPD selects *u* as follows to improve the betweenness and closeness of *t*, when multiple nodes available to be chosen as *u*.

(2) $$u=\underset{u\notin N_{G}(t)}{\text{argmax}}w_{b}\cdot b_{G}(u)+w_{c}\cdot c_{G}(u)+(1-w_{b}-w_{c})\cdot d_{G}(u),$$

where *b_G_*(*u*) and *c_G_*(*u*) denote the betweenness and closeness of *u*, and *w_b_* and *w_c_* are the weights of betweenness and closeness in selecting *u*. *w_b_* (or *w_c_*) is derived by computing the difference of *t*’s betweenness and *ω_b_* (or *t*’s closeness and *ω_c_*) when *t*’s betweenness (or closeness) is smaller than *ω_b_* (or *ω_c_*). Otherwise, *w_b_* (or *w_c_*) is set as 0.

### 3.2 Solution Quality in Threshold Graph

We prove that CRPD obtains the optimal solution for *threshold graphs*, which are similar to many well-known online social networks in terms of important properties like the scale-free degree distribution, short diameter, and clustering coefficient [26, 31, 45].

Definition 2. *A graph G* = (*V*, *E*) *is a threshold graph if there exists a weight function* *f*(·) : *V* → *R and a threshold t_G_*, *such that for any two nodes i, j* ∈ *V, f*(*i*) + *f*(*j*) > *t_G_ if and only if* (*i*, *j*) ∈ *E*.

Theorem 2. *The CRPD algorithm can find an optimal solution of NILD-S when G* = (*V*, *E*) *is a threshold graph, and the running time of CRPD is* $O(|E|\hat{d}+k\hat{d}|V|)$, *where* $\hat{d}$ *is the maximum degree in G* = (*V*, *E*).

With the property of threshold graphs, we prove that CRPD always finds a feasible solution, and the obtained feasible solution is one of the optimal ones in [19].

## 4 INTERVENTION FOR MULTIPLE TARGETS

We formulate the *Network Intervention with Limited Degradation - for Multiple targets* (NILD-M) problem and show the NP-hardness.

Definition 3. *Given a social network G* = (*V*, *E*) *(or G for short), the number of k intervention edges to be added, the LCC degradation threshold τ, the lower bounds on betweenness, closeness and degree* *ω_b_*, *ω_c_*, and *ω_d_*, *and a set of targeted individuals T, the NILD-M problem minimizes the maximal LCC among all nodes in T, i.e.*, $\max_{t\in T}\;LCC_{\bar{G}}(t)$, *by selecting a set of k intervention edges F, such that in the new network* $\bar{G}$, *1)* $LCC_{\bar{G}}(v)-LCC_{G}(v)\leq \tau$ *for any v*, 2) $b_{\bar{G}}(t)>\omega_{b}$ for any *t* ∈ *T*, *3)* $c_{\bar{G}}(t)>\omega_{c}$ *for any t* ∈ *T, and 4)* $d_{\bar{G}}(t)>\omega_{d}$ *for any t* ∈ *T, where* $b_{\bar{G}}(t)$, $c_{\bar{G}}(t)$ *and* $d_{\bar{G}}(t)$ *are the betweenness, closeness, and degree of t in* $\bar{G}$.

Corollary 1. *NILD-M isNP-hard and cannot be approximated within any ratio in polynomial time unless P=NP*.

Corollary 1 follows since NILD-S, the special case of NILD-M when ∣*T*∣ = 1, is NP-hard and cannot be approximated within any ratio in polynomial time unless P=NP in Theorem 1.

## 5 THE OISA ALGORITHM

A naïve approach for NILD-M is to exhaustively search every *k*-edge set spanning the nodes in *T*. However, the approach is not scalable as shown in Section 6. In the following, we first present two baseline heuristics, *Budget Utility Maximization (BUM) and Surrounding Impact Minimization (SIM)* for NILD-M.

### Budget Utility Maximization (BUM).

To intervene the maximal number of individuals within *T*, BUM repeatedly selects the node *u* having the largest LCC without contradicting any constraints and connects *u* to the node *m* with the largest LCC in *T* until *k* edges are selected.

### Surrounding Impact Minimization (SIM).

Without considering the proximity between *m* and *u*, adding (*m*, *u*) sometimes increases the LCCs of their common neighbors. To avoid the above situation, SIM chooses the *u* with the maximum number of hops from *m* in *T* because adding (*m*, *u*) is less inclined to change the LCCs of other neighbor nodes.

In summary, BUM carefully evaluates the LCC of *u* but ignores the structural properties, whereas SIM focuses on the distance between *m* and *u* but overlooks the LCC. Be noted that BUM and SIM are also equipped with MISS to ensure they obtain feasible solutions. Example 2 indicates that their solutions are far from the optimal solution.

**Figure 3:**
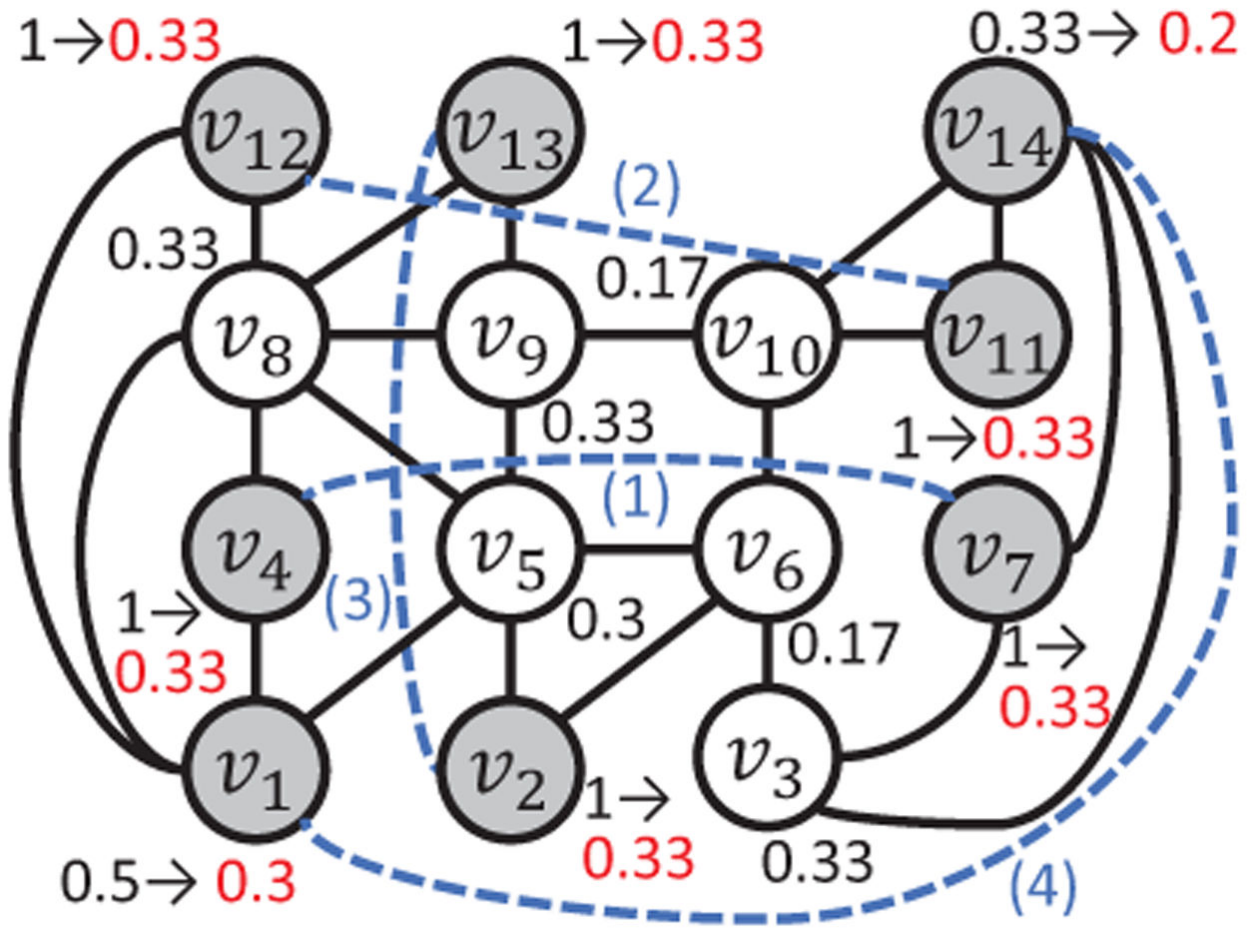
A motivating example of OISA

Example 2. Figure 3 *presents an examplewith k* = 4 *and τ* = 0.15. *G* = (*V*, *E*) *includes 14 nodes and 23 black solid edges, where each node v is labeled aside by its LCC_G_*(*v*) *in dark. The targeted T includes 8 grey nodes with the largest LCC in V, i.e., T* = {*v*_2_, *v*_4_, *v*_7_, *v*_11_, *v*_12_, *v*_13_, *v*_1_, *v*_14_}. *ω_b_*, *ω_c_*, *ω_d_* *are set as 0.02, 0.44, and 3, respectively. The weights W_b_, w_c_, and* (1 – *w_b_* – *w_c_*) *of MISS are set as 0.33, 0.33, and 0.34. For this instance, the maximal LCCs obtained by BUM and SIM are both 1. In contrast, the maximal LCC in the optimal solution is 0.66 acquired by adding the dotted blue edges into F. For each node v whose* $LCC_{\bar{G}}(v)$ *is not equal to* *LCC_G_*(*v*), *its* $LCC_{\bar{G}}(v)$ *are labeled aside in red. The optimal solution effectively lowers the maximal LCC by 34% from BUM and SIM without increasing any node’s LCC*.

Motivated by the strengths and pitfalls of BUM and SIM, we propose *Objective-aware Intervention Edge Selection and Adjustment (OISA)* to jointly consider the LCCs and the network structure with three ideas: 1) *Expected Objective Reaching Exploration (EORE)*, and 2) *Poor Optionality Node First (PONF)*, and 3) *Acceleration of LCC Calculation (ALC)*. EORE finds the minimum number of intervention edges required to achieve any targeted LCC for each candidate solution and sees if it can meet the budget constraint *k*. Given a targeted LCC, PONF carefully adds intervention edges to avoid seriously increasing LCCs for some individuals. A pseudocode of OISA is shown in Algorithm 2.

### 5.1 Expected Objective Reaching Exploration

EORE carefully examines the correlation between the LCC reduction and the number of intervention edges. Lemma 1 first derives the minimum number of required intervention edges *k_G_* for *G* to achieve any targeted LCC. To meet the degree constraint *ω_d_*, the minimum number of edges for each node *t* ∈ *T* is at least *ω_d_* – *d_G_*(*t*).

Lemma 1. *Given a node t of degree d_G_(t), to reduce* $LCC_{\bar{G}}(t)$ *to any targeted LCC l, the minimum number of intervention edges k_t_ is the smallest number satisfying:*

(3) $$\frac{LCC_{G}(t)\times d_{G}(t)(d_{G}(t)-1)}{\left(d_{G}(t)+k_{t})(d_{G}(t)+k_{t}-1\right)}\leq l.$$

*Also, the minimum number of edges k_G_ for G is* 0.5 ·∑_*t*∈*T*_ max{*k_t_*, *ω_d_* – *d_G_*(*t*)}. *(See proof in [*19*].)*

Equipped with Lemma 1, a simple approach is to examine every possible LCC, i.e., $\frac{1}{C(\hat{d},2)}$, $\frac{2}{C(\hat{d},2)}$, where $\hat{d}$ is the maximum degree among all nodes in *G*. However, since adding *k* edges can make intervention for at most 2*k* nodes, it is not necessary to scan all possible LCCs, and EORE thereby only examines a small number of targeted LCCs *l_j_*, where $l_{j}=\frac{j}{C({\hat{d}}_{2k},2)}$ with *j* = 1,2,…, until *l_j_* exceeds the maximal LCC before intervention, and ${\hat{d}}_{2k}\leq \hat{d}$ is the maximum degree among all top-2*k* nodes with the largest LCCs in *T*. OISA skips an *l_j_* if *k_G_* > *k* according to the above lemma.

For any targeted LCC *l_j_*, every node *t* ∈ *T* requires at least max{*k_t_*, *ω_d_* – *d_G_*(*t*)} intervention edges to achieve the targeted LCC *l_j_* and the degree constraint. Thus, OISA stops the edge selection process if there exists a node *t* not able to achieve the above goals when *k* – ∣*F*∣ + *x_t_* < max{*k_t_*, *ω_d_* – *dG*(*t*)}, where *x_t_* is the number of intervention edges incident with *t* in *F*.

### 5.2 Poor Optionality Node First

Recall that BUM ignores the proximity between the two terminal nodes of an intervention edge, and SIM does not examine the LCCs of both terminals and their nearby nodes. Most importantly, both strategies do not ensure the LCC degradation constraint. To address this critical issue, for each targeted LCC, we first propose the notion of *optionality* to identify qualified candidate intervention edges that do not increase the LCC of any individual to more than *τ*.

Definition 4. ***Optionality**. For a target t, the optionality of t denotes the number of nodes in the option set U_t_* ⊆ *T such that for every u_t_* ∈ *U_t_*, *either 1) the hop number from u_t_ to t is no smaller than 3, or 2) u_t_ is two-hop away from t and adding an edge (t, u_t_) does not increase the LCC of any common neighbor by more than τ*.

For the first case, adding an intervention edge (*t*, *u_t_*) does not increase the LCC of any node. For the second case, the LCC degradation constraint can be ensured as long as the LCCs of common neighbors are sufficiently small. Equipped with the optionality, each iteration of PONF first extracts the node *m* with the largest LCC in *T*. If there are multiple candidates for *m* with the same LCC, (e.g., *v*_2_ and *v*_4_ in Figure 3), PONF selects the one with the smallest optionality as *m* so that others with larger optionalities can be employed later. In contrast, if *m* was not selected by now, its optionality tends to decrease later when the network becomes denser and may reach 0, so that the LCC of *m* can no longer be reduced without increasing the LCCs of others, boosting the risk to violate the LCC degradation constraint. The node *u* of the intervention edge (*m*, *u*) is the one with the largest LCC in the option set *U_m_* to reduce the LCCs of both *m* and *u*. PONF also exploits MISS in Section 3.1 to choose *u* according to the differences between *t*’s betweenness and *ω_b_*, and between *t*’s closeness and *ω_c_*.

### 5.3 Acceleration of LCC Calculation

When an edge (*m*, *u*) is added, only the LCCs of *m* and *u* and their common neighbors are likely to change. However, the LCC update cost of *m* is not negligible when the number of neighbors is huge, since it needs to examine whether there is an edge from *u* to each of *m*’s neighbors. The adjacency list of every *m*’s neighbor *v_c_* is required to be inspected even when the LCC of *v_c_* remains the same. To improve the efficiency, ALC avoids examination of every node *v* by deriving its LCC upper bound $\bar{LCC}(v,k)$.

Definition 5. *The LCC upper bound of v after adding k edges is*

(4) $$\bar{LCC}(v,k)=\max_{k_{1}+k_{2}=k}\{\frac{n_{v}+k_{2}+k_{1}\times d_{G}(v)+C(k_{1},2)}{C(d_{G}(v)+k_{1},2)}\},$$

*where d_G_*(*v*) *is the degree of v in G*. *n_v_ is the number of edges between v*’*s neighbors before intervention, n_v_* = *LCC_G_*(*v*) × *C*(*d_G_*(*v*), 2).

Theorem 3. *For an intervention edge set F of size k*, $LCC_{\bar{G}}(v)\leq \bar{LCC}(v,k)$ *holds*.

Proof. Let *k*_1_ and *k*_2_ denote the numbers of intervention edges connecting to *v* and any two neighbors of *v*, respectively. After intervention, *k*_1_ + *k*_2_ ≤ *k*, and $LCC_{\bar{G}}(v)=\frac{n_{v}+k_{2}+y}{C(d_{G}(v)+k_{1},2)}$, where *y* is the number of edges between the new neighbors via the *k*_1_ new edges and the original neighbors of *v* in *G*, and *y* ≤ *d_G_*(*v*) × *k*_1_ + *C*(*k*_1_, 2). Thus, $LCC_{\bar{G}}(v)\leq \frac{n_{v}+k_{2}+d_{G}(v)\times k_{1}+C(k_{1},2)}{C(d_{G}(v)+k_{1},2)}$. Let *k*3 = *k*−*k*_1_−*k*_2_. Then, we obtain $LCC_{\bar{G}}(v)\leq \frac{n_{v}+(k_{2}+k_{3})+k_{1}\times d_{G}(v)+C(k_{1},2)}{C(d_{G}(v)+k_{1},2)}\leq \max_{k_{1}+k_{2}=k}\{\frac{n_{v}+k_{2}+k_{1}\times d_{G}(v)+C(k_{1},2)}{C(d_{G}(v)+k_{1},2)}\}=\bar{LCC}(v,k)$.

**Table:** 

Algorithm 2: The OISA algorithm
$$\begin{array}{cc} & \;\mathrm{Require}:\;G,\;T,\;k,\;\tau ,\;\omega_{b},\;\omega_{c},\;\omega_{d}\\ & \;\mathrm{Ensure}:\;\text{A set}\;F\;\text{of}\;k\;\text{edges to be added, such that the maximal}\\ & \;\;\;\;\text{LCC among nodes in}\;T\;\text{are minimized, while the LCC}\\ & \;\;\;\;\text{increment of any}\;\text{node does not exceed}\;\tau \;\text{and the betweenness,}\\ & \;\;\;\;\text{closeness, and degree of all}\;\text{target nodes exceed}\;\omega_{b},\;\omega_{c},\;\omega_{d}.\\ & \;\;1:j\leftarrow 1\\ & \;\;2:\mathrm{while}\;l_{j}=\frac{j}{C({\hat{d}}_{2k},2)}<\text{the maximum LCC of nodes in}\;G\;\mathrm{do}\\ & \;\;3:\;\;\mathrm{for}\;\text{every}\;t\in T\;\mathrm{do}\\ & \;\;4:\;\;\;\;\text{Calculate}\;k_{t}\;\text{according to Lemma 1}\\ & \;\;5:\;\;\text{Set}\;k_{G}\;\text{as the sum of max}\{k_{t},\omega_{d}-d_{G}(t)\}\;\text{of all}\;t\in T\\ & \;\;6:\;\;\mathrm{if}\;k_{G}<k\;\mathrm{then}\\ & \;\;7:\;\;\;\;F\leftarrow \emptyset \;∕∕\text{Enter the PONF process}\\ & \;\;8:\;\;\;\;\mathrm{for}\;i=1\ldots k\;\mathrm{do}\\ & \;\;9:\;\;\;\;\;\;\text{Choose}\;m\;\text{as the node with maximal LCC in}\;T\\ & \;10:\;\;\;\;\;\;\text{Choose}\;u\;\text{according to Definition 4 and Eq. 2}\\ & \;11:\;\;\;\;\;\;\text{Add}\;(m,u)\;\text{into}\;F\\ & \;12:\;\;\;\;\;\;\text{Recompute nodes}’\;\text{LCC with the acceleration of ALC}\\ & \;13:\;\;\;\;\text{Recod}\;F\;\text{if it reaches a smaller maximal LCC}\\ & \;14:\;\;j\leftarrow j+1\\ & \;15:\mathrm{return}\;\text{The best}\;F\;\text{found} \end{array}$$

According to the above theorem, ALC first derives *LCC_G_*(*v*) and $\bar{LCC}(v,k)$ as a pre-processing step of OISA before intervention. Accordingly, PONF does not update the LCC of a node *v* if the intervention edge neither connects to *v* nor spans *v*’s two neighbors, and *v* is not going to be *m* and *u* in the next iteration since $\bar{LCC}(v,k)$ is smaller than the current maximal LCC potential to be *m* and *u* in the next iteration. Therefore, Theorem 3 enables OISA to effectively skip the LCC updates of most nodes.

Theorem 4. *The time complexity of OISA is O*(*n_l_* × *k* × (*n_m_* × (∣*V*∣ + ∣*E*∣))), *where n_l_ is the number of targeted LCCs, and n_m_ is the number of nodes with the largest LCC. (See proof in* [19].)

## 6 EXPERIMENTAL RESULTS

We evaluate the effectiveness and efficiency of CRPD and OISA by experimentation in Sections 6.1 and 6.2, respectively. Also, to show the feasibility of using OISA in real-world setting, we present an empirical study in Section 6.3.^3^ The study has been inspected by 11 clinical psychologists and professors in the field.^4^

For simulation, since there is no prior work on lowering LCCs while ensuring their betweenness, closeness and degree, we compare the proposed CRPD and OISA with five baselines: 1) Budget Utility Maximization (*BUM*): BUM iteratively adds an edge between a targeted node and the node with the largest LCC, while not violating the constraints; 2) Surrounding Impact Minimization (*SIM*): SIM iteratively adds an edge from a targeted node to the node with the maximal number of hops from it, while not violating the constraints; 3) Enumeration (*ENUM*): ENUM exhaustively finds the optimal solution. 4) Edge Addition for Improving Network Centrality (*EA*) [29]: EA iteratively adds an edge with the largest increment on closeness centrality, and 5) Target-oriented Edge Addition for Improving Network Centrality (*TEA*) [10]: TEA iteratively adds an edge with the largest increment on closeness centrality for the targeted nodes. 6) Greedy algorithm for Dyad scenario (*GD*) [50]: GD iteratively adds an edge with the largest increment on influence score, where the influence score is calculated in a similar way to PageRank. All algorithms are implemented on an HP DL580 G9 server with four Intel Xeon E7-8870v4 2.10 GHz CPUs and 1.2 TB RAM. Six real datasets are evaluated in the experiments. The first one, *CPEP* [16], contains the complete social network of the students in 10 classrooms of several public elementary schools in the US. The other five large real social network datasets, collected from the Web, are *Facebook*, *Flickr*, *Youtube*, *Amazon*, and *Cond-Mat*. Some statistics of datasets used in our experiments are summarized in Table 2. The default *τ*, *ω_b_* and *ω_c_* are set to 0.12, 0.01, and 0.1, respectively.

### 6.1 Evaluation of CRPD for NILD-S

**Figure 4:**
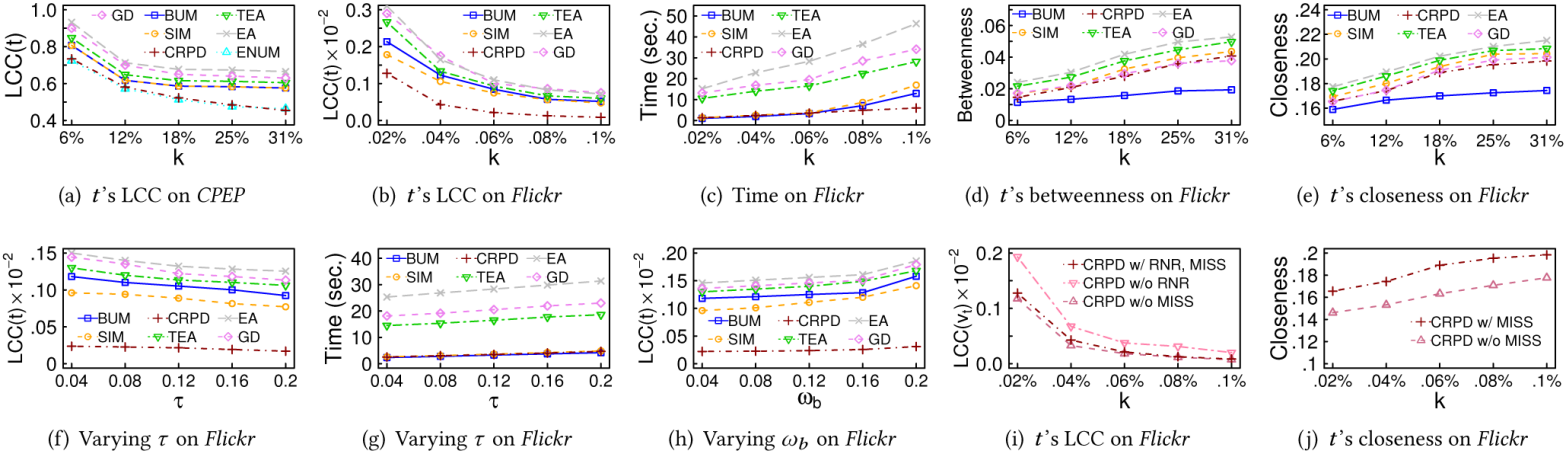
Sensitivity tests for NILD-S

In the following, we first compare CRPD with baselines by varying *k*. For *Facebook* and *Flickr*, ENUM does not return the solutions in two days even when *k* = 10, and thus it is not shown. Figures 4(a)-(e) compare the results of various *k* on *CPEP* and *Flickr* under default *τ*, *ω_b_* and *ω_c_*. For *CPEP*, *k* is set to 6%, 12%, 18%, 25%, and 31% of the number of nodes (i.e., 1, 2, 3, 4, and 5 intervention edges). For *Flickr*, *k* is set to 0.02%, 0.04%, 0.06%, 0.8%, and 0.1% of nodes (i.e., 400, 800, 1200, 1600, and 2000 intervention edges). The t is randomly chosen from the nodes with LCC larger than 0.8, and we report the average of 50 trials. Figures 4(a) and 4(b) show that with *k* increasing, *t*’s LCC decreases as more edges are connected with *t* to reduce its LCC. Also, CRPD outperforms other baselines as it carefully examines the candidates’ structure to avoid increasing the number of edges among *t*’s neighbors. Figure 4(c) shows the running time of OISA is comparable with simple baselines BUM and SIM while achieving better LCC on *Flickr*.

Figures 4(d) and 4(e) show the betweenness and closeness of *t* after adding the intervention edges to *Flickr*. EA and TEA obtain the largest betweenness and closeness, but their maximal LCCs are not effectively reduced. SIM also achieves large betweenness and closeness since it selects the node farthest from *t* as *u* and creates a shortcut between them, but the maximal LCC of SIM does not significantly decrease. GD achieves smaller betweenness and closeness than SIM since it generally selects a node *u* with a large PageRank score, but its improvement on *t*’s betweenness and closeness is smaller than SIM. BUM induces the smallest betweenness and closeness of *t* since it selects *u* with the largest LCCs, and those chosen *u* are inclined to be near each other. In contrast, CRPD achieves comparable performance with EA and TEA, showing that CRPD can effectively improve not only LCC but also other network characteristics.

Next, we conduct a series of sensitivity tests on *τ*, *ω_b_*, and *ω_c_*, and show the component effectiveness. The results on *CPEP* and *Facebook* are similar to *Flickr* and thus not shown here.

#### Varying *τ*.

Figure 4(f) compares LCC of *t* with different *τ* on *Flickr*, under default *ω_b_* and *ω_c_*. As shown, the LCC of *t* decreases because a large *τ* allows more nodes to be candidates and thereby tends to generate a better solution. Figure 4(g) shows that the running time of CRPD and baselines on *Flickr* increases as *τ* grows because more candidates are considered.

#### Varying *ω_b_* and *ω_c_*.

Figure 4(h) compares the LCC of *t* with different *ω_b_* on *Flickr*, where *k* = 0.04% (800 intervention edges). The trend of *ω_c_* is similar to that of *ω_b_* and thus not shown here. LCC of *t* slightly grows with increasing *ω_b_* and *ω_c_*, because large *ω_b_* and *ω_c_* require CRPD and baselines to allocate more edges for the betweenness and closeness, instead of focusing on reducing LCC. Moreover, LCC of *t* obtained by TEA, EA, and CRPD increases slower than one obtained by BUM, SIM and GD, since the edges selected by TEA and EA maximize the closeness centrality while fulfilling the constraints of *ω_b_* and *ω_c_*, whereas the edges from CRPD effectively increase the betweenness and closeness of *t*.

#### Component Effectiveness.

Figure 4(i) compares LCC of *t* obtained by CRPD with and without RNR, and Figure 4(j) compares the closeness of CRPD with and without MISS on *Flickr*. The trend of betweenness is similar to closeness and thereby not shown here. As shown, while CRPD with and without MISS shares similar LCCs, CRPD with MISS achieves a greater closeness because choosing *u* with larger closeness/betweenness tends to increase *t*’s betweenness and closeness. The results of CRPD on *CPEP* and *Facebook* share a similar trend and thus are not shown here.

### 6.2 Evaluation of OISA for NILD-M

In the following, we evaluate the proposed OISA by randomly choosing 20% of nodes in *V* as *T* from the nodes with the top-40% maximum LCCs. The results are the average of 50 trials.

**Figure 5:**
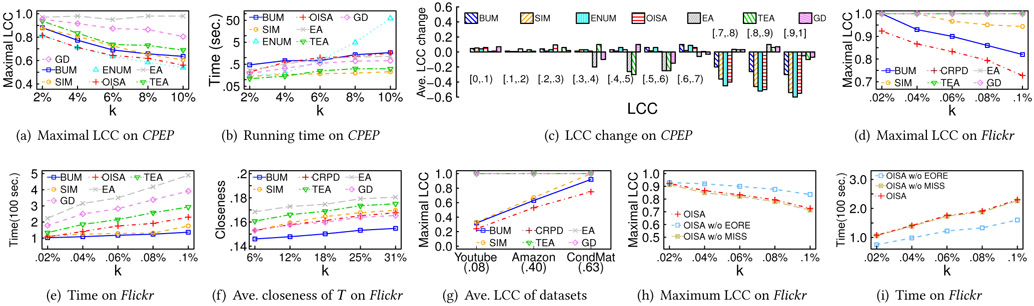
Scalability & sensitivity tests for NILD-M

Figures 5(a)-(c) first compare the effectiveness of all examined approaches on *CPEP* by varying the number of intervention edges *k* (relative to the edge number in *G*). Figure 5(a) indicates that as *k* grows, the maximum LCC generally decreases. OISA and ENUM significantly outperform BUM, SIM, EA, TEA, and GD because EA, TEA and GD are designed to maximize the closeness centrality and influence score, instead of reducing the maximum LCC.Also, BUM only examines the LCCs of the nodes, while SIM ignores LCCs and gives the priority to the intervention edges with the maximum numbers of hops. Figure 5(b) presents the running time of all approaches in the log-scale. OISA and most baselines select *F* within 1 second. In contrast, the running time of ENUM grows exponentially.

To understand the changes of LCCs in different nodes, we take a closer look at the nodes whose LCC potentially changes, i.e., the terminal nodes of the selected edges *F* and their neighbors. Figure 5(c) presents the average LCC change of the nodes in each LCC range after intervention where *k* = 6%. As shown in Figure 5(c), even though BUM outperforms SIM, the average LCC reduction in the range [0.9, 1] is smaller than SIM since BUM may connect nearby nodes and increase the LCCs of the common neighbors. It also explains why the maximum LCC achieved by BUM drops slower than SIM, ENUM, and OISA while *k* increases, i.e., BUM creates lots of targeted nodes with LCCs around 0.6 and 0.7, and BUM needs to make intervention for all of them again to reduce the maximum LCC to become lower than 0.6. In contrast, the behavior of OISA is similar to ENUM in most ranges, and it successfully achieves comparable performance.

Next, Figures 5(d)-(i) compare all approaches except ENUM on *Flickr*, since ENUM does not return any solution in two days even for *k* = 10. The result on *Facebook* is similar and thereby is not shown here. As there are nearly 9000 nodes with LCCs as 1 on *Flickr*, the minimal *k* is 4500 edges, because adding one edge can make intervention for at most two nodes with LCC as 1. Thus, *k* is set to 0.02%, 0.04%, 0.06%, 0.08%, and 0.1% of the number of edges (i.e., 4500, 9000, 13500, 18000, and 22500 edges). Figure 5(d) indicates that OISA significantly outperforms all other baselines under all settings of *k*. BUM is superior to SIM when *k* is small because the farthest node *u* selected by SIM usually results in a small LCC and fewer neighbors, i.e., reducing the LCC of *u* does not help reduce the maximum LCC. Thus, when the number of intervention edges to be added is small, e.g., smaller than the number of targeted nodes in *T*, it is impossible for SIM to reduce the maximum LCC. In contrast, *u* selected by BUM usually has a large LCC, and thus BUM is able to reduce the maximum LCC with the number of intervention edges around half of the minimal *k*.

Figure 5(e) compares the running time. EA considers every possible edge and thus incurs the largest running time. BUM requires the least running time since it only retrieves the nodes with the largest LCC as the terminals of the intervention edges. OISA needs slightly more time but obtains much better solutions since it carefully examines multiple anticipated LCCs, i.e., *l_j_*. Also, OISA takes a longer time on *Flickr* since *Flickr* has much more large-LCC nodes, and it is necessary for OISA to derive the optionality for all these nodes. The running time of EA and TEA grows significantly on *Flickr* than on *CPEP*, as EA, TEA and GD need to examine every candidate edge not in *E*, and *CPEP* is denser than *Flickr*. Figure 5(f) shows that the average closeness of targeted nodes in various *k*. Be noted that the average betweenness of targeted nodes has a similar trend with Figure 5(f) and thus eliminated here. The difference between OISA and baselines is similar to the case of NILD-S.

Next, we conduct a sensitivity test on the average LCC of networks and show the component effectiveness. The results on *CPEP* and *Facebook* are similar to those of *Flickr* and thus not shown here.

#### Varying average LCC.

We evaluate OISA on *Youtube* (average LCC 0.08), *Amazon* (average LCC 0.40), and *Cond-Mat* (average LCC 0.63) with *k* = 0.3% of the number of edges in each network, i.e., *k* = 8963 for *Youtube*, *k* = 2778 for *Amazon*, and *k* = 280 for *Cond-Mat*. Figure 5(g) shows that the maximum LCC is larger when the average LCC of the dataset grows, and OISA outperforms other baselines.

#### Component effectiveness.

Figures 5(h) and 5(i) evaluate the different components in OISA. The result indicates that OISA achieves high maximal LCC with smaller computation time.

### 6.3 Empirical Study

The empirical study aims at evaluating the utility and feasibility of the proposed network intervention algorithm in real-world settings. The study, spanned over two months, included 8 weekly measurements of psychological outcomes among 424 participants, aged between 18 and 25. The participants were university students and employees with 638 pre-existing friendship links at the beginning of the study. Four self-reported standard psychological questionnaires were adopted as indicators, including *Beck Anxiety Inventory* (BAI) [2] for *anxiety, Perceived Stress Scale* (PSS) [8] for *stress, Positive And Negative Affect Schedule* (PANAS) [48] *for emotion*, and *Psychological well-being Scale* (PWS) [39] for *well-being*. In PANAS, there are 12 positive emotion terms and 14 negative emotion terms, and the overall score is the total score of 12 positive emotion terms minus the total score of negative emotion terms. For *anxiety* and *stress*, higher scores indicate higher levels of anxiety and perceived stress, respectively. For *emotion* and *well-being*, higher scores imply better emotion and psychological well-being.

To evaluate the effects of adding friendship links based on different approaches, the participants were randomly assigned to one of the following four groups: three *intervention groups* and one *control group*, with 103 participants in every group.^5^ Participants in the intervention groups were provided with explicit friendship recommendations suggested by OISA and other two baselines, GD and BUM, respectively. Among the five baselines, GD was chosen because it can be applied to propagate health-related information to prevent obesity [50]. BUM was chosen because it performs the best among all baselines in Section 6.2. In the study, *k* was set as 28 for OISA, GD, and BUM, whereas *τ* = 0.12, *ω_b_* = 0.01, *ω_c_* = 0.1, and ∣*T*∣ = 103 for OISA. For OISA, GD, and BUM, the recommended edges were suggested by providing specific instructions for the participants to engage in online chatting. In contrast, participants in the control group received no intervention and explicit instruction to interact with other participants. Each participant was required to provide responses to the four questionnaires every week for a total of eight times in this study.

An important question in the study is *whether the participants accepted the friendship recommendations or not*. To answer this question, participants were also asked the following questions at the end of the study:

Q1. Do you feel happy chatting with this recommended participant?

Q2. After the study ends, are you willing to chat with this participant?

Q3. If possible, are you willing to become a friend of this participant?

For Q1, 80.2% of the participants reported that they felt happy during the chat with the recommended participants. For Q2, 79.9% of the participants replied that they would be willing to chat with the recommended participants even after the study ended. For Q3, 83.9% of the participants reported that they would be willing to become a friend of the recommenced participant. Accordingly participants in this study tended to accept the friend recommendations.

**Figure 6:**
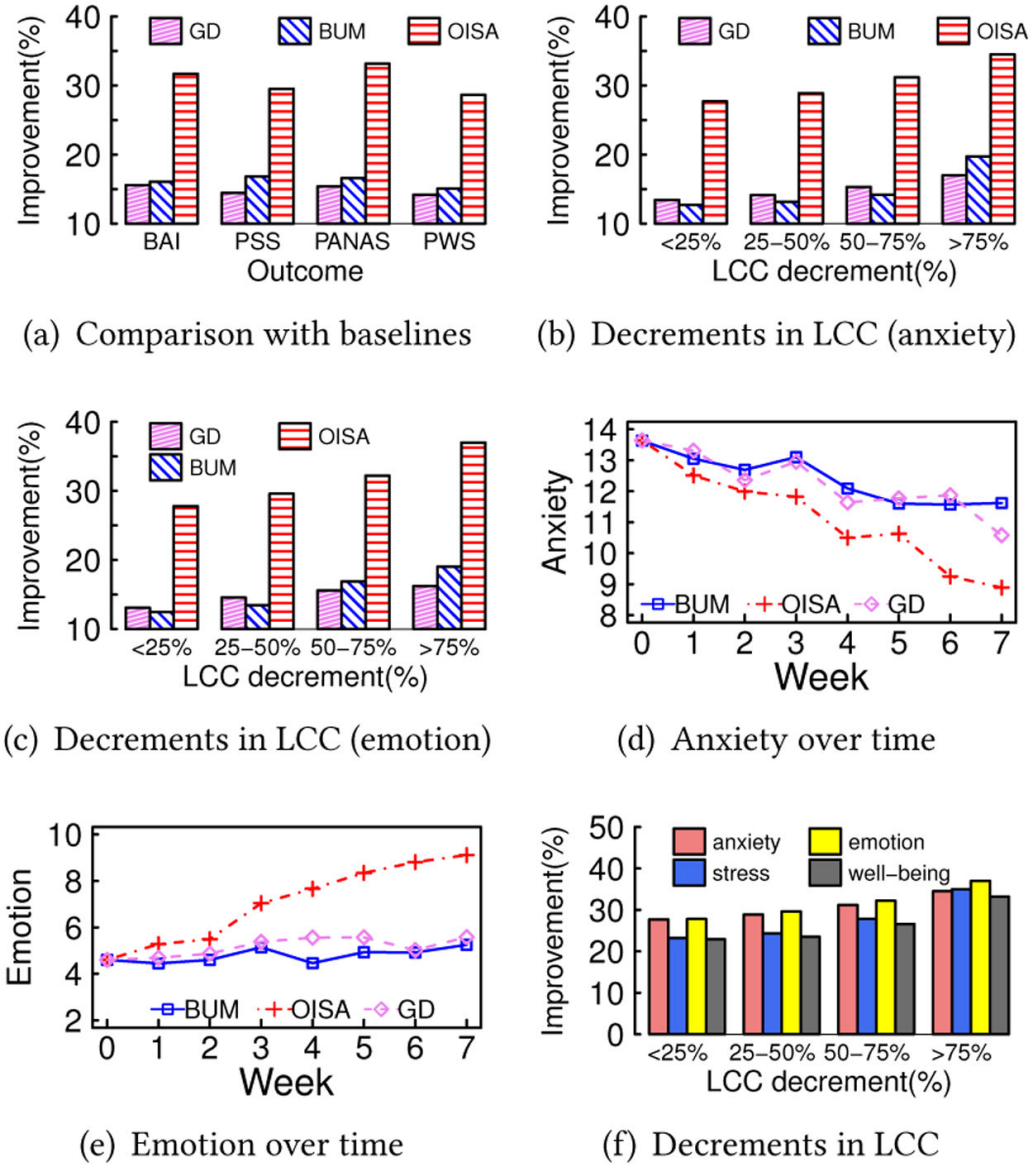
Empirical study results

To evaluate the effects of the intervention, Figure 6 reports the average improvement on each psychological outcome for GD, BUM and OISA. As shown in Figure 6(a), among three intervention groups, OISA outperforms GD and BUM in all four measures, *anxiety, stress, emotion*, and *well-being*. Figures 6(b)-(c) further divide participants into four sub-groups according to the percentage of reduction in LCC.^6^ Figures 6(b)-(c) manifest that greater percentages of reduction in LCC are associated with more significant improvements in the psychological outcomes. It validates that new friendships via OISA is able to improve the psychological outcomes in this study. GD is inferior due to a different goal (i.e., maximizing the social influence score). Figures 6(d)-(e) plot the average scores of anxiety and emotion of participants in the intervention groups of GD, BUM, and OISA over time. As shown in Figures 6(d)-(e), the improvements of OISA is the most significant. In contrast, GD and BUM do not consider multiple network characteristics simultaneously. Figure 6(f), which plots the improvement of OISA for each outcome, also manifests that the improvements on anxiety, stress, and emotion with negative feelings are slightly better than that on well-being with only positive feelings, because brains tend to focus on potential threatening and negative emotions [21]. In this case, the friend candidate recommended by OISA is able to provide social support to the participants.

We also evaluate OISA, GD and BUM separately with mixed effects modeling [20], a statistical technique to examine if the intervention group and control group are statistically different. Specifically, we fitted the model:

(5) $$H_{it}=(\beta_{0}+\beta_{0i})+\beta_{1}\cdot intervention_{i}+\beta_{2}\cdot time\;\;+\beta_{3}\cdot intervention_{i}\cdot time+\epsilon_{it},$$

where *H_it_* represents participant *i*’s emotion at time *t*; *intervention_i_* denotes whether participant *i* is in the control group (*intervention_i_* = 0) or the intervention group (*intervention_i_* = 1); *time* represents the number of weeks elapsed since the study started. The study starts at time 0. *β*_0_ is a group intercept term representing the predicted outcome for the control group at time 0. *β*_0*i*_ is the participant *i*’s deviation in intercept relative to *β*_0_, which is a random effect in intercept at time 0. *β*_1_ – *β*_3_ are the regression weights associated with *intervention_i_*, *time* and their interaction, respectively. Specifically, *β*_1_ indicates the difference in predicted psychological outcomes between the control group and intervention group at time 0; *β*_2_ represents the estimated amount of change in emotion in the control group for each week of elapsed time (i.e., the “placebo effect”, or the improvements in psychological outcomes shown by the control group). Finally, *β*_3_ is the most important, i.e., the regression coefficient for the *intervention_i_* · *time* interaction reveals the estimated difference in the amount of change in outcomes reported by the intervention group *relative to* the control group for each week of elapsed time. If *β*_3_ is statistically significantly different from 0, OISA (or other baselines) is able to change the participants’ health outcomes with time substantially more than what is expected in the control group. Finally, *ϵ_it_* represents the residual error in the negative emotion that cannot be accounted for by other terms.

Table 3 presents the results of model fitting of OISA. Estimates with *p*-values smaller than 0.05 are identified as statistically significantly different from 0. Thus, the two values of *β*_0_, the predicted psychological outcomes of the control group at time 0, are estimated to be significantly different from 0. The term *β*_1_ is not significantly different from 0 for all the four outcomes, which validates our random assignment procedure – indicating that the intervention and control group do not differ substantially in their anxiety, stress, emotion and well-being scores at time 0. Also, the control group does not show substantial changes in all the four outcomes with time, as *β*_2_ is not significant. Moreover, the values of *β*_3_ are negative and significantly different from zero for anxiety and stress, indicating that the intervention group shows statistically greater decreases in anxiety and stress. Similarly, the values of *β*_3_ are positive and significantly different from zero for emotion and well-being, indicating that the intervention group shows statistically greater increase in emotion and well-being. In other words, the intervention group, whose friendship recommendation is suggested by OISA, shows statistically greater improvement for all the four outcomes. Also, the more extreme test statistic value (*t*-value) and its corresponding *p*-value, associated with the outcomes, suggest that the intervention have a more systematic effect (i.e., less uncertainty or smaller standard error) [5]. Also, Table 4 reveals that the estimates and *p*-values of *β*_0_–*β*_3_ for BUM and GD are not statistically significant.^7^

Lastly, inspection of these results by 11 clinical psychologists and professors^4^, is carried out to observe the behavioral implications behind the scores. The psychologists and professors are asked to the following question in Likert scale: Is the network intervention help improve the participants’ outcomes? Comparing their evaluation among intervention and control groups, the results indicate that 82% of psychologists and professors agree that the recommendation of OISA is the most effective; while only 9% of them agree for GD and BUM. The above results lead to consistent conclusion with the study – the intervention is therapeutic and positive enough to be brought into clinical consideration.

## 7 CONCLUSION

Even though research has suggested the use of network intervention for improving psychological outcomes, there is no effective planning tool for practitioners to select suitable intervention edges from a large number of candidate network characteristics. In this paper, we formulate NILD-S and NILD-M to address this practical need. We prove the NP-hardness and inapproximability, and propose effective algorithms for them. Experiments based on real datasets show that our algorithms outperform other baselines in terms of both efficiency and effectiveness; empirical results further attest to the practical feasibility and utility of using the OISA algorithm for real-world intervention purposes.

## Figures and Tables

**Table 1:** Summary of notations

Term	Meaning	Term	Meaning
*G*	original network	*F*	selected intervention edges
*t*, *T*	targeted individual(s)	$\bar{G}$	network after intervention
*N_G_*(*v*)	*v*’s neighbors in *G*	*u*	node to be connected with *t*
*b_G_*(*v*)	*v*’s betweenness in *G*	$\hat{d}$	maximum degree in *G*
*c_G_*(*v*)	*v*’s closeness in *G*	*R*	nodes removed by CRPD baseline
*d_G_*(*v*)	*v*’s degree in *G*	*r*	a removed node in *R*
*LCC_G_*(*v*)	*v*’s LCC in *G*	*w_b_*	weights of betweenness in MISS
*ω_b_*	lower bound of betweenness	*w_c_*	weights of closeness in MISS
*ω_c_*	lower bound of closeness	*w_d_*	weights of degree in MISS
*ω_d_*	lower bound of degree	*f*(·)	weight func. in threshold graph
*τ*	LCC degradation constraint	*k_G_*	lower bound on the num. of
*k*	# of intervention edges		needed edges in EORE
*n_v_*	# of edges among *v*’s neighbors	*l_j_*	targeted LCC to be tried in EORE

**Table 2:** Dataset statistics

dataset	∣*V*∣	∣*E*∣	ave. LCC	dataset	∣*V*∣	∣*E*∣	ave. LCC
CPEP [16]	226	583	0.71	*Youtube* [38]	1.1M	3.0M	0.08
*Facebook* [46]	60.3K	1.5M	0.22	*Amazon* [38]	33.5K	92.6K	0.40
*Flickr* [28]	1.8M	22.6M	0.25	*Cond-Mat* [38]	23.1K	93.5K	0.63

**Table 3:** Results comparing the over-time changes in outcomes of the intervention (OISA) and control groups

		Estimates	Std errors	df	*t*-values	*p*-values
*Anxiety*	*β*_0_	13.64	1.17	320.64	11.70	<0.0001***
	*β*_1_	−0.69	1.65	323.10	−0.42	0.6763
	*β*_2_	−0.09	0.15	1082.86	−0.59	0.5532
	*β*_3_	−0.61	0.21	1085.94	−2.97	0.0030**
*Stress*	*β*_0_	14.84	0.32	364.78	46.02	<0.0001***
	*β*_1_	0.76	0.46	367.97	1.66	0.0982
	*β*_2_	0.07	0.04	1083.49	1.56	0.1187
	*β*_3_	−0.19	0.06	1087.00	−2.95	0.0033**
*Emotion*	*β*_0_	4.60	1.14	298.29	4.05	<0.0001***
	*β*_1_	−0.08	1.61	300.36	−0.05	0.9580
	*β*_2_	−0.25	0.13	1082.17	−1.85	0.0640
	*β*_3_	0.96	0.19	1084.98	5.09	<0.0001***
*Well-being*	*β*_0_	74.54	1.07	241.65	69.73	<0.0001***
	*β*_1_	0.09	1.51	242.67	0.06	0.9545
	*β*_2_	−0.18	0.09	1081.12	−1.95	0.0515
	*β*_3_	0.45	0.13	1082.87	3.41	0.0007***

**Table 4:** Results comparing the over-time changes in outcomes of the intervention (GD, BUM) and control groups

		GD		BUM	
		Estimates	*p*-values	Estimates	*p*-values
*Anxiety*	*β*_0_	13.64	<0.0001***	13.63	<0.0001***
	*β*_1_	−0.48	0.7990	−3.08	0.0752
	*β*_2_	−0.09	0.5780	−0.08	0.5827
	*β*_3_	−0.29	0.2190	−0.26	0.2615
*Stress*	*β*_0_	14.84	<0.0001***	14.83	<0.0001***
	*β*_1_	0.09	0.0840	0.85	0.0654
	*β*_2_	0.07	0.0970	0.07	0.1105
	*β*_3_	0.07	0.2450	−0.05	0.4454
*Emotion*	*β*_0_	4.60	0.0002***	4.60	<0.0001***
	*β*_1_	0.36	0.8394	2.42	0.1262
	*β*_2_	−0.25	0.0648	−0.25	0.0613
	*β*_3_	0.38	0.0638	0.29	0.1291
*Well-being*	*β*_0_	74.54	<0.0001***	74.54	<0.0001***
	*β*_1_	−0.35	0.8322	1.00	0.5161
	*β*_2_	−0.18	0.0636	−0.18	0.0529
	*β*_3_	0.25	0.1496	0.26	0.0537

## Footnotes

1 According to the study on 2844 junior high school students over three years [41], the decrement of depression rates is significantly correlated with the reduction of LCCs with Pearson Correlation Coefficient as 0.3.

2 Adding (*v*_1_, *v*_4_) to *F* only changes the LCCs of *v*_4_ and *v*_5_, where $LCC_{\bar{G}}(v_{4})$ – *LCC_G_*(*v*_4_) = 0.33 – 0.4 < 0.05 and $LCC_{\bar{G}}(v_{5})$ – *LCC_G_*(*v*_5_) = 0.2 – 0.17 < 0.05.

3 The IRB number is 10710HE072.

4 They are from California School of Professional Psychology, Taipei City Government Community Mental Health Center, National Taipei University of Nursing and Health Science, National Taiwan University etc.

5 Before the study started, participants in the four groups were requested to fill the questionnaires to ensure that there were no statistically significant differences for those questionnaires between every pair of groups.

6 Results of pressure, and well-being are similar and thus not shown here.

7 The standard errors, degree of freedom, and *t*-values are shown in [19].
